# Three-dimensional growth dynamics of the liver in the human fetus

**DOI:** 10.1007/s00276-015-1437-4

**Published:** 2015-02-03

**Authors:** Michał Szpinda, Monika Paruszewska-Achtel, Alina Woźniak, Mateusz Badura, Celestyna Mila-Kierzenkowska, Marcin Wiśniewski

**Affiliations:** 1 Department of Normal Anatomy, The Ludwik Rydygier Collegium Medicum in Bydgoszcz, The Nicolaus Copernicus University in Toruń, Łukasiewicza 1 Street, 85-821 Bydgoszcz, Poland; 2 Department of Medical Biology, The Ludwik Rydygier Collegium Medicum in Bydgoszcz, The Nicolaus Copernicus University in Toruń, Karłowicza 24 Street, 85-092 Bydgoszcz, Poland

**Keywords:** Liver length, Liver transverse diameter, Liver sagittal diameter, Liver growth, INTERGROWTH-21st Project, Centiles, Regression curves

## Abstract

**Purpose:**

The fetal liver is indubitably the earliest and the most severely affected organ by abnormal fetal growth. The size of the fetal liver assessed by three-dimensional ultrasonography is indispensable in determining the status of fetal growth, nutrition and maturity, and in the early recognition and monitoring fetal micro- and macrosomias. The aim of the present study was to measure the human fetal liver length, transverse and sagittal diameters to establish their age-specific reference intervals, the 3rd, 10th, 50th, 90th, and 97th smoothed centile curves, and the relative growth of the liver calculated for the 50th centile.

**Materials and methods:**

Using anatomical, digital (NIS-Elements AR 3.0, Nikon) and statistical methods (one-way ANOVA test for paired data and post hoc RIR Tukey test, Shapiro–Wilk test, Fisher’s test, Student’s *t* test, the Altman-Chitty method), length, transverse and sagittal diameters of the liver for the 3rd, 10th, 50th, 90th, and 97th centiles were assessed in 69 human fetuses of both sexes (32 males and 37 females) aged 18–30 weeks, derived from spontaneous abortions or stillbirths.

**Results:**

No male–female differences (*P* > 0.05) concerning the three parameters studied were found. During the study period, the fetal liver increased tri-dimensionally: in length from 19.51 ± 1.02 to 39.65 ± 7.05 mm, in transverse diameter from 29.44 ± 3.73 to 53.13 ± 5.31 mm, and in sagittal diameter from 22.97 ± 3.79 to 43.22 ± 5.49 mm. The natural logarithmic models were found to fit the data with gestational age (*P* < 0.001) in the following five cutoff points: 3rd, 10th, 50th, 90th and 97th centiles. The values of liver parameters in relation to gestational age in weeks were calculated by the following logarithmic regressions: *y* = −82.778 + 35.752 × ln(age) ± *Z* × (−2.778 + 0.308 × age) for liver length, *y* = −123.06 + 52.668 × ln(age) ± *Z* × (3.156 + 0.049 × age) for liver transverse diameter, and *y* = −108.94 + 46.052 × ln(age) ± *Z* × (−0.541 + 0.188 × age) for liver sagittal diameter. For the 50th centile, at the range of 18–30 weeks, the growth rates per week were gradually decreasing from 1.93 to 1.21 mm for length, from 2.85 to 1.79 mm for transverse diameter, and from 2.49 to 1.56 mm for sagittal diameter of the liver (*P* < 0.05). During the study period both the length-to-transverse diameter ratio and the sagittal-to-transverse diameter ratio of the liver changed little, attaining the values of 0.71 ± 0.11 and 0.87 ± 0.12, respectively.

**Conclusions:**

The fetal liver does not reveal sex differences in its length, transverse and sagittal diameters. The fetal liver length, transverse and sagittal diameters grow logarithmically. The regression equations for the estimation of the mean and standard deviation of liver length, transverse and sagittal diameters allow for calculating any desired centiles according to gestational age. The three-dimensional evolution of the fetal liver follows proportionately. The age-specific reference intervals for evolving liver length, transverse and sagittal diameters constitute the normative values of potential relevance in monitoring normal fetal development and screening for disturbances in fetal growth.

## Introduction

The fetal liver is indubitably the earliest and most markedly affected organ by abnormal fetal growth [30]. The growing liver is extremely sensitive and responsive to maternal glucose levels, so its length considerably increases among mothers with gestational diabetes when compared to normal pregnancies [12]. In clinical practice, direct ultrasonic measurement of the liver right lobe in utero was found to be considerably useful than indirect measurement of abdominal circumference [30]. Therefore, the size of the fetal liver assessed by three-dimensional ultrasonography is indispensable in determining the status of fetal growth, nutrition and maturity, and particularly in the early recognition and monitoring both fetal micro- and macrosomias [7, 15, 19, 31]. In fact, a decreased size of the fetal liver is typical of intrauterine growth retardation [14], while its increased size is a good indicator of fetal macrosomias, Rh isoimmunization, erythroblastosis, Hb Bart’s disease, congestive heart failures, and intrauterine infections [1, 3, 13, 14, 20]. Despite the widespread use of 3D-ultrasound and MRI worldwide, conventional autopsy still remains the gold reference standard in the quantitative evaluation of fetal organs [5]. This means that from the clinical perspective, visceral measurements and growth curves obtained anatomically are as relevant as ultrasonic measurements.

In the medical literature concerning hepatic morphometric parameters in the human fetus [1, 6, 9, 17, 29, 31], we failed to find complete information about liver growth dynamics with relation to its length, transverse and sagittal diameters. To date, however, the only anatomical research on the quantitative analysis of the fetal liver performed by Albay et al. [1] has focused on its mean values for height, width and thickness in particular three trimesters and in term fetuses, but with no growth dynamics. On the base of ultrasonography, proportionate growth dynamics against gestational age were modelled for liver length by some authors [9, 17, 29], and for liver length, transverse and sagittal diameters by Chang et al. [6], only. In the light of the recently published INTERGROWTH-21st Project [18], to supplement fragmentary information on liver dimensions in the human fetus, our objectives were to establish:

- reference intervals for dimensions (length, transverse and sagittal diameters) of the fetal liver at consecutive gestational ages (age-specific reference intervals),
- the 3rd, 10th, 50th, 90th, and 97th smoothed centile curves for the liver length, transverse and sagittal diameters vs. gestational age, and
- the relative growth of the fetal liver calculated for the 50th centile (length-to-transverse diameter ratio, sagittal-to-transverse diameter ratio).

## Materials and methods

**Fig. 1:**
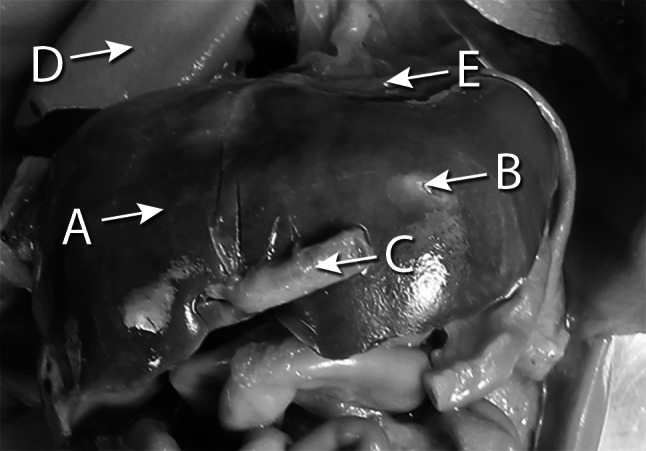
The liver in situ (frontal projection) in a female fetus at the age of 19 weeks: *A* right lobe of liver, *B* left lobe of liver, *C* umbilical vein, *D* right lung, *E* abdominal diaphragm. Of note, unlike in the adults, the fetal liver is nearly symmetrical

The examinations were carried out in Department of Anatomy of the Ludwik Rydygier Collegium Medicum in Bydgoszcz. The study encompassed a group consisting of 69 autopsied human fetuses of both sexes (32 males, 37 females) aged 18–30 weeks of Caucasian origin (Table 1), derived from spontaneous abortions or stillbirths in the years 1989–1999. According to the INTERGROWTH-21st Project, the fetal ages in weeks were fine-tuned by the following criteria: the fetal crown-rump length (gestational age), known date of the beginning of the last maternal menstrual period (amenorrhea age), and known values of the five fetal anthropometric measurements: head circumference, bi-parietal diameter, occipitofrontal diameter, abdominal circumference, and femur length assessed by early second trimester ultrasound scan (ultrasound age) [4, 18, 23–28, 31]. The crown-rump length was measured with the use of a flexible caliper from the top of the head (crown) to the bottom of the buttocks (rump) of the fetus in its natural C-shaped posture [16]. As a prerequisite, the eligible sample was built by rejection of fetuses from diabetic mothers or multiple pregnancies, and fetuses affected by congenital and chromosomal anomalies. Furthermore, the fetuses studied could not suffer from growth retardation, because the correlation coefficient between the gestational age based on the crown-rump length and that calculated by either the amenorrhea age or ultrasound age reached the value *R* = 0.99 (*P* < 0.001) for the whole sample. So, the fetuses included could be considered as normal. Legal and ethical considerations were approved by the University Research Ethics Committee (reference: KB 161/2013). After having been immersed in 10 % neutral buffered formalin solution for 12–24 months [4, 11, 16, 23–28], the fetuses were anatomically dissected through median and transverse laparotomy under tenfold magnification using a stereoscope with Huygens ocular (Fig. 1). After opening the abdominal cavity, the hepatic ligaments, abdominal diaphragm, inferior vena cava and structures at the porta hepatis were cut off, and then the liver was removed out. Every isolated liver with a millimeter scale was placed vertically to the optical lens axis, recorded in both the anterior and superior projections with the use of Canon 550D camera, digitalized to TIFF images, and afterward assessed using digital image analysis.

**Table 1:** Age, number and sex of the fetuses studied

Gestational age (weeks)	*n*	Sex		Crown-rump length (mm)			
		Male	Female	Mean	SD	Min	Max
18	4	3	1	138.3	5.5	131.0	143.0
19	6	4	2	151.5	3.6	145.0	155.0
20	7	3	4	162.4	3.2	159.0	167.0
21	7	5	2	173.7	3.6	170.0	180.0
22	6	1	5	185.2	3.1	181.0	190.0
23	6	4	2	199.5	3.9	195.0	204.0
24	10	2	8	210.0	3.8	205.0	214.0
25	5	2	3	216.0	2.2	215.0	220.0
26	3	1	2	230.3	4.6	225.0	233.0
27	4	2	2	239.5	3.1	235.0	242.0
28	7	1	6	251.0	2.6	247.0	253.0
30	4	4	0	264.0	1.2	263.0	265.0

For anatomists dealing with fetuses the most objective information for establishing fetal ages is the crown-rump length, when compared to the known data of the beginning of the last maternal menstrual period or to ultrasonic measurements of head circumference, bi-parietal diameter, occipitofrontal diameter, abdominal circumference, and femur length

**Fig. 2:**
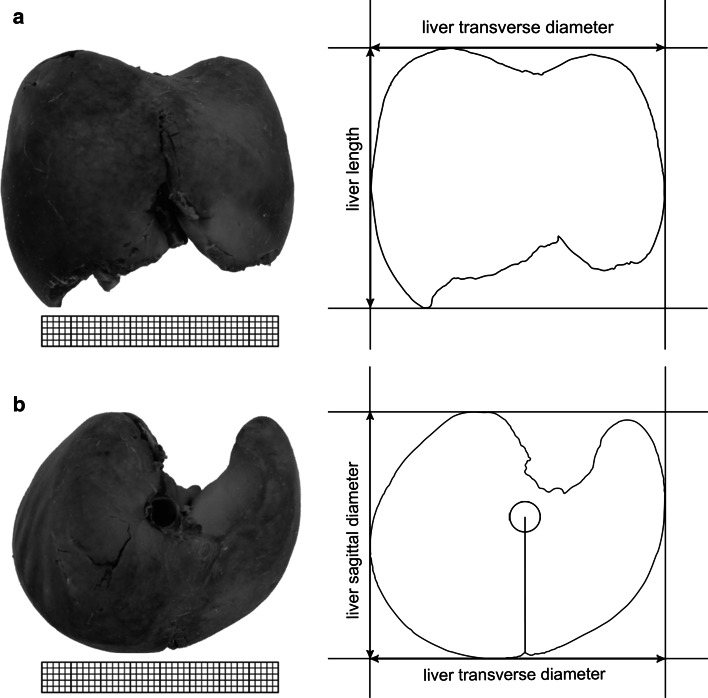
Measurements of the liver in a female fetus aged 23 weeks in the frontal (**a**) and superior (**b**) projections

It should be emphasized that a valid objective semi-automatic software package (NIS Elements AR 3.0, Nikon) was used for measuring the length, transverse and sagittal diameters of the fetal liver, with the greatest accuracy in measuring the selected dimensions to the nearest 0.01 mm. For every fetus the following three measurements (Fig. 2) and two calculations of the liver were performed:

1. length in mm, corresponding to the greatest vertical distance of the liver, from its superior to inferior border on the right lobe, measured in the anterior projection,
2. transverse diameter in mm, corresponding to the greatest horizontal distance of the liver, from its right to left border, measured in the anterior projection,
3. sagittal diameter in mm, corresponding to the greatest sagittal distance of the liver, from its anterior to posterior border on the right lobe, measured in the superior projection,
4. length-to-transverse diameter ratio, and
5. the sagittal-to-transverse diameter ratio.

In an incessant effort to minimize measurement and observer bias, all measurements were carried out by one researcher (M.P.A.). Each measurement was performed three times under the same conditions but at different times, and the average was incorporated into numerical data. In the present study the statistical program Statistica 10 was used. The differences between the repeated measurements, as the intra-observer variation were assessed by the one-way ANOVA test for paired data and post hoc RIR Tukey test. The numerical data were tested for normality of distribution by the Shapiro–Wilk test and for homogeneity of variance by Fisher’s test. The statistical analysis was started by assessing the probability of appearance of statistically significant differences in values with relation to sex with the use of Student’s *t* test for unpaired variables. The fetuses studied were separated into 12 one-week intervals not equally distributed with respect to fetal age. Furthermore, some 1-week intervals did not represent adequate samples, including either 4 (fetuses aged 18, 27, and 30 weeks) or 3 (fetuses aged 26 weeks) specimens, only. So, the first four intervals (18–21 weeks), the successive three intervals (22–24) and the last five intervals (25–30 weeks) were separately grouped. Therefore, to examine the possible sex differences, firstly we checked differences between the following three age groups: 18–21 (*n* = 24), 22–24 (*n* = 22), and 25–30 (*n* = 23) weeks, and secondly for all the fetuses, irrespective of fetal age. To check whether or not significant differences existed with age, the one-way ANOVA test for unpaired data and post hoc RIR Tukey test were used for the 3 aforementioned age groups. The establishment of charts of the liver length, transverse and sagittal diameters followed the Altman-Chitty method [16, 18]. By doing so we established the mean, standard deviation, and the five centiles (3rd, 10th, 50th, 90th, and 97th) for each parameter at each gestational age. After that, the 3rd, 10th, 50th, 90th, and 97th smoothed centile curves for the liver length, transverse and sagittal diameters vs. gestational age were modelled. The relative growth of the fetal liver calculated for the 50th centile was expressed as the length-to-transverse diameter ratio, and the sagittal-to-transverse diameter ratio. Differences were considered significant at *P* < 0.05.

## Results

In total, 69 fetuses met the eligibility criteria and were enrolled for morphometric and statistical analysis. No statistically significant differences were found in the evaluation of intra-observer reproducibility of the liver measurements (*P* > 0.05). Since the statistical analysis of liver parameters revealed no sex differences (*P* > 0.05), the analysis could be applied to the entire group. The growth curves of best fit for the plot for each parameter studied vs. gestational age turned out to be statistically significant (*P* < 0.001). Natural logarithmic models were found to fit the data.

**Fig. 3:**
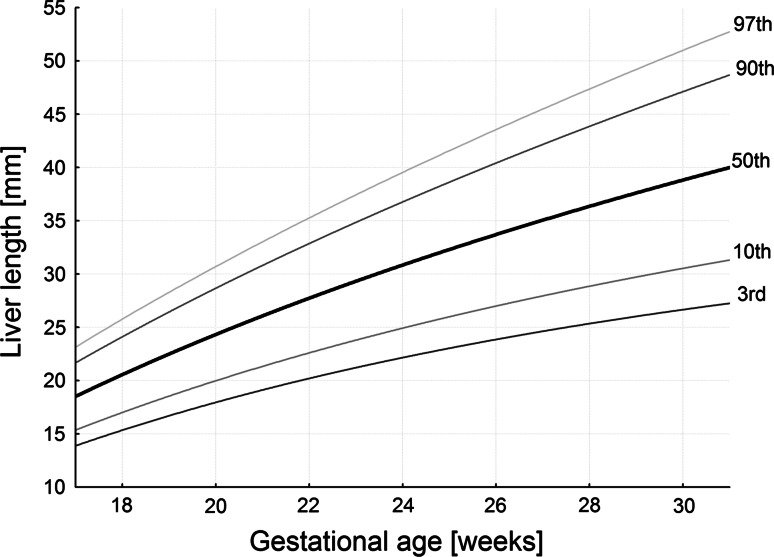
3rd, 10th, 50th, 90th and 97th smoothed centiles for liver length vs. fetal age

**Fig. 4:**
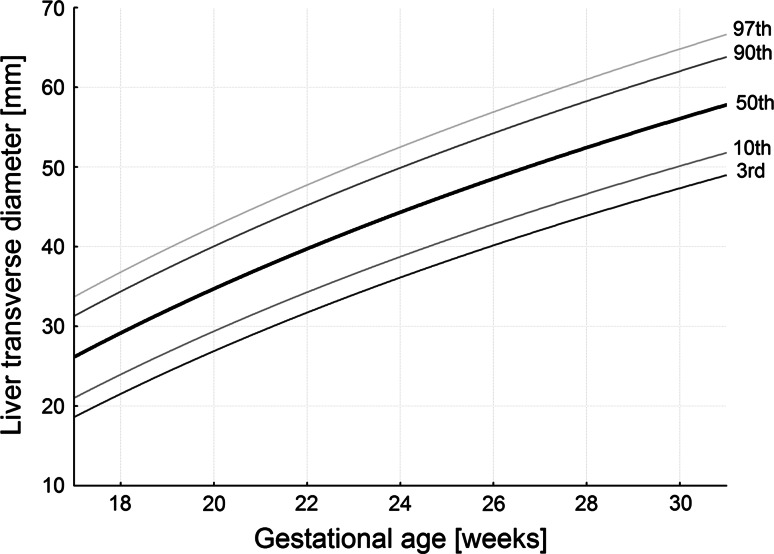
3rd, 10th, 50th, 90th and 97th smoothed centiles for liver transverse diameter vs. fetal age

**Fig. 5:**
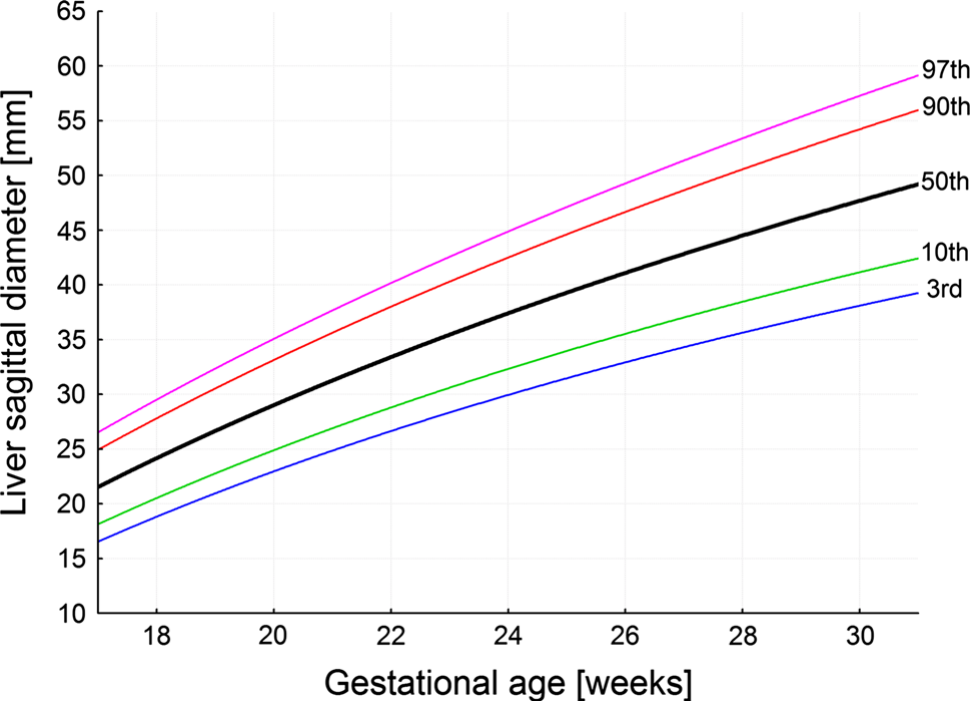
3rd, 10th, 50th, 90th and 97th smoothed centiles for liver sagittal diameter vs. fetal age

The numerical data are presented in Table 2. The values of liver length ranged from 19.51 ± 1.02 mm for the 18-week group to 39.65 ± 7.05 mm for the 30-week group of gestation. During that time the liver transverse diameter incrementally increased from 29.44 ± 3.73 to 53.13 ± 5.31 mm. Furthermore, the liver sagittal diameter took the values of 22.97 ± 3.79 mm at the age of 18 weeks and 43.22 ± 5.49 mm in fetuses aged 30 weeks. The best fitting curves for the three measures (liver length, transverse and sagittal diameters) obtained were presented in the following five cutoff points: 3rd, 10th, 50th, 90th and 97th centiles (Figs. 3, 4, 5). Their corresponding regression equations for the estimation of the mean and SD (in mm) of liver length, transverse and sagittal diameters according to gestational age (in weeks) have been displayed in Table 3. The particular centiles have been calculated as mean ± *Z* × SD. In terms of statistics, the value of *Z* depends on a particular centile, and constantly equals −1.88 for the 3rd centile, −1.28 for the 10th centile, 0 for the 50th centile, +1.28 for the 90th centile, and +1.88 for the 97th centile. The values of liver parameters in relation to gestational age in weeks were calculated by the following logarithmic regressions: *y* = −82.778 + 35.752 × ln(age) ± *Z* × (−2.778 + 0.308 × age) for liver length (Fig. 3), *y* = −123.06 + 52.668 × ln(age) ± *Z* × (3.156 + 0.049 × age) for liver transverse diameter (Fig. 4), and *y* = −108.94 + 46.052 × ln(age) ± *Z* × (−0.541 + 0.188 × age) for liver sagittal diameter (Fig. 5). After that for the 50th centile, we calculated the growth rate per week for each parameter studied. At first, by substituting fetal ages of 18, 19, 29 and 30 weeks in the three logarithmic models we calculated for 50th centile the real values for liver length, transverse and sagittal diameters in the four aforementioned weeks. Next, the two growth rates per week for each parameter were obtained as the difference between particular values in fetuses aged 19 and 18 weeks, and 30 and 29 weeks, respectively. At the range of 18–30 weeks for the 50th centile, the growth rates per week were gradually decreasing from 1.93 to 1.21 mm for length, from 2.85 to 1.79 mm for transverse diameter, and from 2.49 to 1.56 mm for sagittal diameter of the liver (*P* < 0.05).

**Table 2:** Length, transverse and sagittal diameters of the fetal liver

Gestational age (weeks)	*n*	Anterior projection				Superior projection	
		Liver length (mm)		Liver transverse diameter (mm)		Liver sagittal diameter (mm)	
		Mean	SD	Mean	SD	Mean	SD
18	4	19.51	1.02	29.44	3.73	22.97	3.79
19	6	20.64	3.24	30.43	1.65	26.44	3.17
20	7	26.79	4.05	34.08	4.25	29.57	4.68
21	7	25.95	3.35	37.55	6.15	31.25	3.54
		↓(*P* < 0.01)		↓(*P* < 0.01)		↓(*P* < 0.01)	
22	6	27.91	6.40	39.62	3.00	31.78	1.74
23	6	32.89	5.34	43.27	4.45	36.71	3.16
24	10	31.13	4.14	44.40	4.24	38.34	2.89
25	5	35.53	6.08	50.83	2.51	39.60	4.37
26	3	31.47	2.51	47.78	1.06	44.14	4.17
27	4	36.81	6.59	50.63	5.92	38.48	5.33
28	7	33.51	3.80	50.81	9.52	45.94	6.01
30	4	39.65	7.05	53.13	5.31	43.22	5.49

Means for liver length, transverse and sagittal diameters in columns between the three age groups of 18–21, 22–24, and 25–30 weeks differ significantly

**Table 3:** Equations for the estimation of the mean and SD (in mm) of liver length, transverse and sagittal diameters according to gestational age (in weeks)

Parameter	Regression equation	
	Mean	SD
Liver length	−82.778 + 35.752 × ln(age)	−2.778 + 0.308 × age
Liver transverse diameter	−123.06 + 52.668 × ln(age)	3.156 + 0.049 × age
Liver sagittal diameter	−108.94 + 46.052 × ln(age)	−0.541 + 0.188 × age

All ln are natural logarithms, age means gestational age

**Fig. 6:**
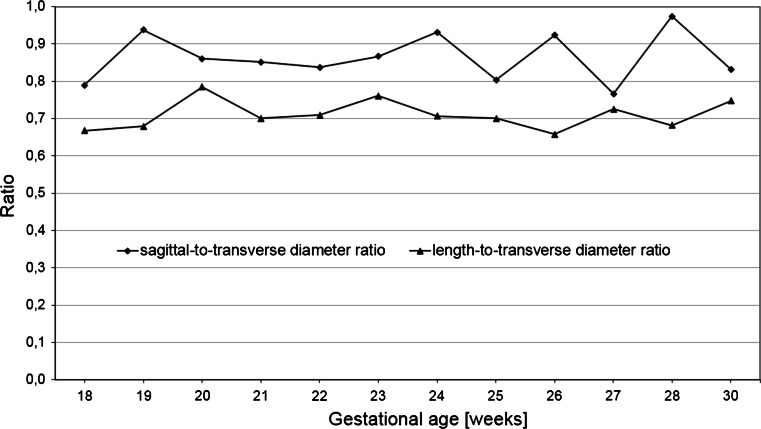
Length-to-transverse diameter ratio and sagittal-to-transverse diameter ratio of the liver calculated for the 50th centile vs. gestational age

During the duration of the study period both the length-to-transverse diameter ratio and the sagittal-to-transverse diameter ratio of the liver changed little, attaining the values of 0.71 ± 0.11 and 0.87 ± 0.12, respectively (Fig. 6).

## Discussion

The present study describes a cross-sectional interpretation of the three dimensions (length, transverse and sagittal diameters) of the growing liver in human fetuses. In total, 69 autopsied formalin-fixed fetuses at the age of 18–30 weeks were enrolled for final analysis. So, the sample was relatively numerous, comparable and representative of fetal age. In fact, the fetuses studied might potentially be smaller for gestational age. However, the assumption that fetuses could suffer from intrauterine growth retardation was unambiguously disapproved, because the correlation coefficient between the gestational age based on the crown-rump length and that calculated by either the amenorrhea age or ultrasound age reached the value *R* = 0.99 (*P* < 0.001) for the whole sample. In clinical practice, a reliable estimate of gestational age up to 14 weeks is confirmed by ultrasound measurement of fetal crump-rump length. As recommended by the Fetal Growth Longitudinal Study, part of the International Fetal and Newborn Growth Consortium for the 21st Century (INTERGROWTH-21st) Project, in women commencing antenatal care at less than 14 weeks and 0 days of gestation until birth, a combination of known values of the five fetal ultrasonic measurements: head circumference, bi-parietal diameter, occipitofrontal diameter, abdominal circumference, and femur length behooves estimation of accurate fetal ages [4, 18, 23–28, 31]. The Fetal Growth Longitudinal Study prospectively assessed fetal growth from 14 to 42 weeks of gestation in 4,321 mothers who were at low risk of intrauterine growth restriction (optimal health and nutritional maternal wellbeing, satisfactory antenatal care, singletons without congenital malformations) in eight geographically diverse urban populations [18]. Of note, the ultrasound measurements were taken specifically and the method was rigorous and implemented across all study sites with the same specially adapted ultrasound equipment to allow blinding of measurements. As a result, the 3rd, 5th, 10th, 50th, 90th, and 97th centile curves vs. gestational age for the five primary ultrasound growth measures were produced, as the international standards for fetal growth.

As far as formalin-fixed fetuses are concerned, it should be emphasized that formalin fixation little (0.5–1.0 %) influences both size and shape of parenchymal viscera preserved in situ in the sealed thoracic and abdominal cavities [4, 11, 16, 23–28]. On the contrary, any isolated organ subjected to formalin fixation is prone to artifacts as a result of tissue shrinkage. We decided not to assess the liver weight, because the 12–24 months formalin preservation could certainly gain the weight and density of fixed organs, even by 20–25 % for the liver [8]. Our results were based on plausible direct measurements, instead of deduced, extrapolated through a series of indirect measurements. All the parameters measured were exact and clearly definable. We opine that our empirical results could be considered as veracious because both the material studied and the methods used were apposite.

In the present study, no significant male–female differences concerning all the three morphometric parameters of the fetal liver were found, in accordance with other reports [1, 6, 13, 21]. Thus, we did not attempt to further separate our findings with relation to sex.

It is explicitly apparent that the fetal liver growth is three-dimensional with simultaneous evolution of length, transverse and sagittal diameters. To date, however, except for one anatomical study by Albay et al. [1] and one ultrasonographic research by Chang et al. [6], reference data for liver dimensions in the human fetus have focused on the liver length only. Albay et al. [1] basing on 121 autopsied fetuses aged 9–40 weeks examined the height, width, and thickness of the liver, that were tantamount to its length, transverse and sagittal diameters, respectively. The liver length gradually increased from 13 ± 2 mm in fetuses aged 9–12 weeks (1st trimester) through 25 ± 8 mm in fetuses aged 13–25 weeks (2nd trimester) and 41 ± 7 mm in fetuses aged 26–37 weeks (3rd trimester) to 47 ± 8 mm in fetuses aged 38–40 weeks (full term). Some authors [12, 17, 22, 29, 30] 2-D ultrasonically studied the liver length to present its normal range for every week of pregnancy. Vintzileos et al. [30] ascertained that the mean liver length incrementally increased from 27.3 to 59.0 mm in fetuses aged 20 and 40 weeks, respectively. Roberts et al. [22] showed that in 350 normal pregnancies, the fetal liver length crept up in a probably parabolic manner, from 12.5 mm in fetuses aged 13 weeks to 51 mm in fetuses aged 40 weeks. In the ultrasonographic group of Mirghani et al. [12], consisting of 31 fetuses aged 21–24 weeks, the liver length ranged from 30 to 33 mm. Phatihattakorn et al. [17] presented sonographically derived results from liver length measurements of 750 fetuses at the age of 13–40 weeks, ranging from 12.59 ± 2.08 to 60.06 ± 3.49 mm. These findings coincide with those reported by Tongprasert et al. [29], who published the results on normal liver length in 640 fetuses from 14 to 40 weeks of gestation. During that time, the liver length increased from 9.9 to 51.9 mm for the 5th percentile, from 15.8 to 57.7 mm for the 50th percentile, and from 21.7 to 63.6 mm for the 95th percentile [29]. As reported by Kuno et al. [9], the liver length increased from 25 mm for the 21-week fetus to 58 mm for the 38-week fetus. In the present study, we found the liver length to increase from 19.51 ± 1.02 mm in the 18-week fetus to 39.65 ± 7.05 mm in the 30-week fetus. The meticulous comparative analysis of numerical data revealed that our anatomical results concerning the liver length were in secure consensus with the ultrasonic findings reported by Vintzileos et al. [30], Roberts et al. [19, 22] and Kuno et al. [9]. On the contrary, the means for liver length obtained by Mirghani et al. [12] were smaller by 1–3 %, and those reported by Phatihattakorn et al. [17] and Tongprasert et al. [29] were greater by 4–7 % when compared to our findings. We suggest that the possible differences may be attributed to either anatomical varieties or racial reasons.

In the anatomical material of Albay et al. [1], the transverse diameter of the fetal liver attained the following values: 19 ± 3, 39 ± 12, 67 ± 11, and 82 ± 9 mm for the three successive trimesters of gestation and full term fetuses, respectively. The liver sagittal diameter in the fetus grew from 11 ± 1 mm in the 1st trimester, through 18 ± 5 mm in the 2nd trimester and 26 ± 5 mm in the 3rd trimester to 31 ± 8 mm at full-term fetuses. Regrettably, these authors did not present the means of transverse and sagittal diameters for particular weeks. Moreover, the ultrasonic study reported by Chang et al. [6] showed only two scattergrams for transverse and sagittal diameters, without any numerical data. Finally, our results present the liver transverse and sagittal diameters, changing from 29.44 ± 3.73 to 53.13 ± 5.31 mm, and from 22.97 ± 3.79 to 43.22 ± 5.49 mm, respectively. So, for the forenamed reasons, we could not compare our age-specific intervals for transverse and sagittal diameters of the fetal liver with the findings obtained by Albay et al. [1] and Chang et al. [6]. This has considerably limited our discussion on this subject.

To date, assessment of precise growth dynamics for the growing liver has featured in only a few publications [6, 9, 17, 29, 30]. The growth models divulged by these authors are all alike, presenting a linear relationship between parameters studied and fetal age. In a linear function the rate of growth remains constant throughout the study period. Its value (*a*) precisely corresponds with the coefficient accompanying the fetal age (age), according to the linear formula *y* = *a* × (age) + *b*. According to some authors [2, 11, 22, 30], the fetal liver length grew at a rate of 1.0–1.2 mm per week up to 28 weeks of gestation, and at 1.70–1.76 mm per week after 28 weeks. Phatihattakorn et al. [17] demonstrated a proportionate increase in liver length throughout gestation, that followed in accordance with the linear function: liver length (mm) = 1.528 × (age) − 5.676. Similarly, Tongprasert et al. [29] found the best regression model for the liver length as liver length (mm) = 1.61 × (age) − 6.750 (*R* ^2^ = 0.94; *P* < 0.05). In the 3-D sonographic study of 14 appropriate-for-gestational-age fetuses, reported by Kuno et al. [9], the optimal model for liver length was as follows: liver length (mm) = 1.74 × (age) − 9.47 (*R* ^2^ = 0.858; *P* < 0.001). Therefore, having fastidiously performed the comparative analysis of growth dynamics for liver length, constructed by Phatihattakorn et al. [17], Tongprasert et al. [29] and Kuno et al. [9], we emphasize the affinity between the three growth rates, i.e. 1.528 mm per week, 1.61 mm per week, and 1.74 mm per week, respectively. To date, only Chang et al. [6] with the use of three-dimensional ultrasound studied the growth dynamics of the liver in 55 normal singleton fetuses aged 20–30 weeks with relation to liver length, transverse and sagittal diameters. The scattergrams reflected the following linear functions: *y* = 1.191 × (age) + 0.658 (*R* = 0.58; *P* < 0.001) for liver length, *y* = 2.370 × (age) − 15.897 (*R* = 0.78; *P* < 0.001) for transverse diameter, and *y* = 1.520 × (age) + 4.593 (*R* = 0.56; *P* < 0.001) for sagittal diameter. This means that the growth rate for liver length was much slower (1.191 mm per week) than those reported by the three aforementioned authors. An increase in transverse and sagittal diameters ensued more energetically, when compared to that of liver length, with the following growth rates: 2.370 mm per week and 1.520 mm per week, respectively. In the present study, the numerical data have been presented in a similar manner as the INTERGROWTH-21st Project data [18], including the fitted 3rd, 10th, 50th, 90th, and 97th smoothed centile curves. The length, transverse and sagittal diameters of the fetal liver did not generate linear functions on nomograms. In fact, we substantiated that the best-fit growth models were the following natural logarithmic functions: *y* = −82.778 + 35.752 × ln(age) ± *Z* × (−2.778 + 0.308 × age) for liver length, *y* = −123.06 + 52.668 × ln(age) ± *Z* × (3.156 + 0.049 × age) for liver transverse diameter, and y = −108.94 + 46.052 × ln(age) ± *Z* × (−0.541 + 0.188 × age) for liver sagittal diameter. It is noteworthy that the natural logarithmic function *y* = ln(*x*) is one-to-one (for each *y* there is one and only one *x*), continuous, and increasing. Besides, it indicates a declining rate of change, expressed precisely by a concave down graph, more and more deviating from the linear function *y* = *x* with advanced fetal age. That is because in the material under examination, the growth rates per week for the 50th centile were gradually decreasing from 1.93 to 1.21 mm for length, from 2.85 to 1.79 mm for transverse diameter, and from 2.49 to 1.56 mm for sagittal diameter of the liver (*P* < 0.05) in fetuses aged 18 weeks and 30 weeks, respectively. Therefore, the nomograms presented in the material under examination emphasize a much more extensive growth rate for liver length only in younger fetuses than that presented by all other authors. On the contrary, in older fetuses, the growth rate of liver length was affinitive to that found by Chang et al. [6], and much slower to those presented by Phatihattakorn et al. [17], Tongprasert et al. [29], and Kuno et al. [9]. As far as the transverse and sagittal diameters of the fetal liver are concerned, the only nomograms constructed by Chang et al. [6] support a regular increase in transverse diameter by 2.370 mm per week, and in sagittal diameter by 1.520 mm per week. This growth rate for liver transverse diameter (2.370 mm per week) was comparable to our findings (from 2.85 mm in the 18-week fetus to 1.79 mm in the 30-week fetus). On the contrary, the growth rate for liver sagittal diameter (1.520 mm per week) presented by Chang et al. [6] turned out to be slower to our results (from 2.49 mm in the 18-week fetus to 1.56 mm in the 30-week fetus).

We extrapolated that notwithstanding an absolute increase in the three values studied, the spatial growth of the liver ensued in a relatively proportionate manner. The three-dimensional evolution in the mean length, transverse and sagittal diameters of the fetal liver followed proportionately as 0.71:1:0.87.

Having elucidated the normative growth of liver length, transverse and sagittal diameters in the human fetus, we would like to accentuate the importance of the measurements in the material under examination, because the reader should be endowed with relevant data that are useful for distinguishing abnormal from normal fetal development. Due to the best-fit growth models for the mean and SD (Table 3) for each measure (liver length, transverse and sagittal diameters), readers can calculate any desired centiles according to gestational age. Of note, the value of *Z* always equals −1.88 for the 3rd centile, −1.28 for the 10th centile, 0 for the 50th centile, +1.28 for the 90th centile, and +1.88 for the 97th centile. Murao et al. [14] showed that the fetal liver was considerably small compared to other organs in intrauterine growth retardation. The fetal liver length was found to be below average in 18 % [30] or even 30 % [9, 12, 19] of small-for-gestational-age fetuses. Vintzileos et al. [30] reported on 8 fetuses with severe erythroblastosis fetalis correlated with excessive increases in fetal liver length by 5 mm and over per week. As reported by Roberts et al. [22], enlargement of the fetal liver in isoimmunized pregnancies substantively correlated with increased liver hemopoiesis, and liver length proved to be a conducive indicator of the degree of fetal anemia. Moreover, fetal transfusion and correction of fetal anemia influenced a decrease in fetal liver length. Besides, a considerable increase in liver dimensions in the fetus is a good indicator of the severity, the clinical outcome in pregnancies with Rh isoimmunization, Hb Bart’s disease, congestive heart failures, certain metabolic diseases, tumors, and even intrauterine infections [1, 3, 13, 14, 20]. Roberts et al. [21] ascertained that in fetuses at the age of 18 weeks and over the liver length was 12 % greater in diabetic pregnancies, with relation to normal pregnancies. These authors substantiated that knowledge of the normal range for the fetal liver length is beneficial to diagnose and follow-up both gestational diabetes and hepatomegaly. Mirghani et al. [12] found the mid-trimester fetal liver length to be significantly greater (36 vs. 31 mm) in pregnant women with gestational diabetes mellitus. The measurement of fetal liver length in the diabetic pregnancy is reproducible and of outmost relevance as a parameter for monitoring the effectiveness of treatment in diabetic pregnancies [2, 10].

In our opinion, the main advantages of the present study result in a unique fetal material, objective digital-image analysis, the appropriate statistical analysis, and completely novel growth patterns for the growing liver. The main limitation of the present study seems to be a relatively narrow fetal age, ranging from 18 to 30 weeks of gestation. Had we been able to collect a larger fetal sample size with fetuses younger than 18 weeks and older than 30 weeks, this would considerably hone the growth curves obtained. In nature, all autopsy examinations include retrospective design without prospective ultrasound quality control. Because of this, a disadvantage may be that our findings have been presented as if describing a developmental sequence in one fetus. Therefore, it is rather a populational perspective than a true representation of growth in itself. Furthermore, in our research, measurements were conducted by a single observer in a blind fashion. Besides, our results lack inter-observer variability, because all numerical data have been controlled by one researcher.

In summary, the present paper attempts to cogently extend the existing literature relating to development of the fetal liver in human fetuses. We confer that the crucial findings in our study are that the growth dynamics for the liver length, transverse and sagittal diameters do not follow proportionately, but indicate a disparate fashion, according to logarithmic regressions. The three novel nomograms constructed in the present study may potentially be useful in monitoring normal fetal development and in the diagnosis, monitoring and treatment of severe fetal pathologies that affect the liver in the intrauterine period.

## Conclusions

1. The fetal liver does not reveal sex differences in its length, transverse and sagittal diameters.
2. The fetal liver length, transverse and sagittal diameters grow logarithmically.
3. The regression equations for the estimation of the mean and standard deviation of liver length, transverse and sagittal diameters allow for calculating any desired centiles according to gestational age.
4. The three-dimensional evolution of the fetal liver follows proportionately.
5. The age-specific reference intervals for evolving liver length, transverse and sagittal diameters constitute the normative values of potential relevance in monitoring normal fetal development and screening for disturbances in fetal growth.
